# A distant perspective on how the “past” affects the “present”: the impact of early environmental unpredictability on impulsive consumption

**DOI:** 10.3389/fpsyg.2025.1578234

**Published:** 2025-06-04

**Authors:** Shijin Sun, Zhengyang Hou, Jiahui Guo, Qian Tian

**Affiliations:** Department of Psychology, School of Social Development and Public Policy, Fudan University, Shanghai, China

**Keywords:** impulsive buying behavior, early environmental unpredictability, resource scarcity, life history strategy, consumer behavior

## Abstract

**Introduction:**

With the growing convenience of shopping and the rise of consumerism, impulsive buying has become increasingly prevalent, sometimes leading to harmful consequences. Unlike traditional research focusing on proximal product-related factors, this study investigates the distal influence of early environmental unpredictability on impulsive buying behavior, grounded in life history theory. Additionally, the moderating role of resource scarcity is examined from a broader societal perspective.

**Methods:**

A 2×2 between-subjects factorial experimental design was employed, involving 161 participants. The independent variables were early environmental unpredictability (low vs. high) and perceived resource scarcity (scarcity vs. normal). Participants were randomly assigned to one of the four experimental conditions to explore the relationship between early environmental factors and impulsive consumption tendencies.

**Results:**

The findings revealed that: (1) early environmental unpredictability significantly and positively predicts impulsive buying behavior; and (2) this predictive effect is amplified under conditions of perceived resource scarcity.

**Discussion:**

These results highlight the lasting impact of early life environments on consumer behavior and suggest that resource scarcity can exacerbate impulsive consumption. The study offers practical insights for reducing impulsive buying in modern society and contributes to interdisciplinary understanding in psychology and behavioral economics.

## 1 Introduction

In today’s consumer society, the phenomenon of impulse buying is becoming increasingly prevalent and has attracted widespread attention. Initially, scholars considered impulse buying merely as a type of unplanned purchasing (Clover, 1950). As research has progressed, impulse buying has been recognized as behavior occurring under external stimuli without prior purchasing plans (Beatty and Ferrell, 1998). Moreover, research has expanded to include the cognitive, emotional, and behavioral components of impulse buying. Studies indicate that impulse buyers experience more intense emotional fluctuations (Weinberg and Gottwald, 1982) and that their behavior often appears sudden and uncontrollable (Rook and Fisher, 1995). This study defines impulse buying as an irrational purchasing behavior where consumers pursue immediate satisfaction and make decisions on the spot, accompanied by strong emotional impulses and minimal evaluative judgment. A deeper exploration of the mechanisms and influencing factors of impulse buying is crucial for understanding consumer behavior, guiding rational consumption, and formulating marketing strategies.

The measurement of impulse buying primarily employs methods such as questionnaires, free recall, and situational simulation choice techniques. Questionnaires assess consumers’ impulse buying behaviors through specific questions, like “I often buy products without thinking,” and are the most commonly used method in marketing research (Rook and Fisher, 1995). The free recall method allows participants to recall and record past impulse buying behaviors based on a given definition, though it can be subject to subjective biases (Rook and Fisher, 1995). Situational simulation choice methods involve constructing virtual shopping scenarios and options, enabling participants to make choices in hypothetical situations, thus revealing their tendencies toward impulse buying (Rook, 1987). This study combines questionnaires and situational simulation choice methods to more comprehensively measure and simulate impulse buying behavior.

Concerning the influencing factors of impulse buying, research generally covers three aspects: product characteristics, the consumer environment, and individual characteristics. Firstly, from the product perspective, higher prices usually prompt consumers to engage in more rational thinking, reducing the likelihood of impulse buying (Lin and Chuang, 2005), while the diversity of product categories attracts novelty-seeking consumers, prompting impulse purchases (Sharma et al., 2010). In the consumer environment, time pressure may reduce the opportunities for impulse purchases (Badgaiyan and Verma, 2015). High-quality mall services can also increase consumers’ willingness to buy (Tendai and Crispen, 2009). From the standpoint of individual characteristics, individuals with weaker self-regulation are more prone to impulse buying behavior (Roberts and Manolis, 2012), while factors such as gender, cultural background, and educational level also affect this behavior (Zhang et al., 2018). Additionally, an individual’s impulsive traits are closely related to their purchasing behavior (Turkyilmaz et al., 2015). Moreover, financial literacy has been explored as a factor in consumption behavior, with studies suggesting that higher financial literacy may correlate with increased spending, potentially reflecting more conscious rather than impulsive decisions (Rodriguez et al., 2024). Additionally, financial literacy is linked to sustainable consumer behavior, emphasizing its role in informed financial decision-making (Muñoz-Céspedes et al., 2021). These factors together form a complex network affecting consumers’ impulse buying behavior, providing critical context for designing measurement tools and interpreting research findings.

Despite the extensive research on proximal factors such as product attributes, environmental cues, and individual traits, there remains a significant gap in understanding the distal influences on impulsive consumption, particularly those rooted in early life experiences and long-term environmental conditions. Existing studies often focus on immediate triggers of impulse buying, overlooking how foundational developmental factors shape consumption patterns over time (Beatty and Ferrell, 1998; Sharma et al., 2010). This limitation highlights the need to explore variables such as early environmental unpredictability, which, according to life history theory, may fundamentally influence adaptive behaviors including consumption decisions (Belsky et al., 1991). Furthermore, the interaction between early environmental conditions and situational stressors like resource scarcity is underexplored, yet critical, as it may amplify or mitigate impulsive tendencies under varying economic pressures (Griskevicius and Kenrick, 2013). Therefore, introducing early environmental unpredictability and resource scarcity as independent variables in this study is essential to provide a more comprehensive understanding of impulsive consumption from a distal perspective, addressing the deeper, often overlooked origins of such behavior.

Unpredictability describes the fluctuating nature of an individual’s developmental environment over time, particularly the uncertainty in the temporal and spatial distribution of resources during early life (Proffitt Leyva and Hill, 2018). During the formative years of life (ages 0–14), the stability of the nurturing environment and access to survival resources are crucial, as their variability directly reflects the unpredictability of the environment (Belsky et al., 2012). According to life history theory—an essential concept in evolutionary psychology—individuals raised in unstable environments often exhibit more impulsive behaviors. Life history theory posits that individuals, faced with limited resources in their growth environments, must continually balance tasks related to survival and reproduction, thereby developing strategies that are most adaptive to their surroundings (Belsky et al., 1991; Mittal et al., 2015). Early environmental unpredictability prompts changes in adaptive behaviors, such as early maturation and earlier reproduction times in response to family economic hardships or frequent parental conflicts. This unpredictable childhood environment facilitates adaptive behavioral changes to better cope with chaotic circumstances. Previous studies have shown that individuals living in unpredictable environments are more likely to exhibit aggressive and risk-taking behaviors in adulthood, typically perform poorly academically (Chang et al., 2019), and are associated with antisocial behaviors in adulthood (Zuo et al., 2018), as well as impulsive and risk-taking behaviors (Griskevicius et al., 2011). Mittal’s research further confirmed the relationship between early environmental unpredictability and impulsive behaviors, finding that individuals raised in affluent families displayed lower impulsivity and immediate gratification tendencies compared to those from low-income families (Mittal and Griskevicius, 2014). This study, grounded in life history theory, explores the origins of impulsive consumption, specifically how early environmental unpredictability affects individuals’ resource allocation decisions, thereby influencing impulsive consumption behaviors.

Differences in early-life environments influence how individuals handle adversity in adulthood. The sensitization model suggests that while adults from different early environments behave similarly under normal conditions, their responses diverge under adversity. Resource scarcity, or the lack of production and living materials, leads individuals to take measures to preserve and acquire resources when they perceive a shortage; however, this behavioral tendency decreases when resources are abundant (Chang, 2007). Studies indicate that individuals from unpredictable environments are more sensitive to environmental stressors and more prone to exhibit impulsive behaviors (Mittal and Griskevicius, 2014). Research by Hamilton et al. found that individuals from better early environments tend to save, whereas those from poorer environments are more inclined toward immediate consumption (Hamilton et al., 2019). Differences are not apparent when resource perceptions are stable, but they become evident during fluctuations. In times of resource scarcity, individuals are also more likely to exhibit behaviors such as overspending and excessive borrowing (Griskevicius and Kenrick, 2013). It can be hypothesized that resource scarcity may affect impulsive consumption behaviors differently depending on individuals’ early environments. Lower threat levels of resource scarcity correlate with lower impulsive consumption; higher threat levels increase impulsive consumption. Early environmental unpredictability heightens the propensity for impulsivity under resource scarcity. This study selects social and occupational resource scarcity as representatives of external environmental pressures to explore this phenomenon.

In summary, the existing research on consumption behavior primarily focuses on proximal factors, with less emphasis on distal factors. Therefore, it is essential to innovatively explore new ideas regarding impulsive consumption behaviors, specifically clarifying their relationship with early environmental unpredictability. From a distal perspective, this approach involves a deeper investigation into the underlying reasons for impulsive consumption behaviors. Additionally, in situations of resource scarcity, do individuals proactively reduce their propensity toward impulsive consumption? Do individuals with similar early environmental conditions exhibit different patterns of impulsive consumption under conditions of resource scarcity versus abundance? Based on these considerations, this study aims to explore the impact of early environmental unpredictability on impulsive consumption and to examine the different manifestations of impulsive consumption behaviors among individuals from various early environments when triggered by resource scarcity. The research hypotheses are as follows:

*H1*: Early environmental unpredictability positively predicts impulsive consumption behavior.

*H2*: Individuals with high early environmental unpredictability exhibit increased tendencies toward impulsive consumption under conditions of social environmental resource scarcity.

## 2 Methods

### 2.1 Research design

This study utilized a 2 × 2 between-subjects design, manipulating perceptions of resource scarcity (scarcity group vs. normal group) and early environmental unpredictability (high unpredictability group vs. low unpredictability group). The dependent variable was the level of impulsive consumption.

### 2.2 Participants

Based on the statistical methods applicable to this study, using Gpower 3.1.9.6, a total sample size of 64 (Faul et al., 2007) was required to achieve 80% statistical power at a significance level of *α* = 0.05 and an effect size (*d* = 0.4). A total of 183 participants were randomly recruited, and after data processing, 161 participants remained (86 males, 75 females). Participants meeting basic eligibility criteria (e.g., no prior participation in the pre-experiment, provided informed consent) were randomly assigned to either the resource scarcity perception group (*n* = 74) or the normal resource perception group (*n* = 87) before any manipulation. No pre-assessment of perceived resource scarcity was conducted; manipulation effectiveness was confirmed post-assignment via an independent samples *t*-test (see Section 2.3.2). Post-data collection, participants were categorized into high (*n* = 87) or low (*n* = 74) early environmental unpredictability groups based on the mean score (*M* = 36.47, SD = 8.05) of the respective questionnaire. In the 2 × 2 factorial design, the distribution across groups was: high unpredictability/scarcity (*n* = 41), high unpredictability/normal (*n* = 46), low unpredictability/scarcity (*n* = 33), and low unpredictability/normal (*n* = 41).

The studies involving human participants were reviewed and approved by the Ethics Committee of a certain University in Shanghai (FDU-SSDPP-IRB-2024-2-103, February 1, 2024). They were conducted according to the ethical standards established in the 1964 Declaration of Helsinki and its subsequent amendments. All participants in the study provided informed consent, which includes permission to publish their data (all participants in this study were adults).

### 2.3 Materials

#### 2.3.1 Early environmental unpredictability

The revised Early Environmental Unpredictability Questionnaire by Luo et al. (2020) was used. This questionnaire includes three dimensions: family socio-economic status, living environment unpredictability, and parental emotional unpredictability. Items such as “The rules set by parents often change,” “Parents often moved me from one place to another,” and “Compared to my peers, my life was more affluent” were included. The questionnaire consists of 14 items, and participants responded on a 5-point scale ranging from 1 (completely disagree) to 5 (completely agree). The Cronbach’s alpha coefficient for this scale in the current study was 0.83.

#### 2.3.2 Manipulation of perceived social resource scarcity

The manipulation of perceived social resource scarcity was based on the priming paradigm from Griskevicius and methods adapted from Hill et al., involving issues like employment difficulties to evoke the participants’ perception of resource scarcity (Griskevicius et al., 2013; Hill et al., 2012).

In the resource scarcity condition, experimental materials included excerpts from social media articles describing employment difficulties and public grievances in the current year of 2022 and how “involvement” was formed in today’s social context. In the normal resource condition, the materials were excerpts from articles recalling the good life around 2017 and 2018 and the employment prospects in various industries before the saturation of the internet economy era. The total word count of both passages was controlled to be similar.

After reading the materials, participants were required to answer two questions related to the content to enhance familiarity with the material. Then, they responded to four questions about their perception of resource scarcity, such as “Based on the above materials, do you think there is a shortage of social resources this year?” using a Likert seven-point scale.

Both experimental and control groups read the two social media article excerpts. An independent sample t-test was conducted to evaluate the scores on the material used to prime social resource scarcity perception. The results showed that the scores for the resource scarcity primed group (*N* = 74, *M* = 19.80, SD = 3.38) were significantly higher than those for the normal resource primed group (*N* = 87, *M* = 15.69, SD = 2.88), *t* = 8.33, *p* < 0.001, confirming successful manipulation of the experimental materials.

#### 2.3.3 Measurement of impulsive consumption

To measure impulsive consumption, the situational simulation method was employed, requiring participants to make shopping decisions from five options across four shopping scenarios. The situational simulation paradigm involves presenting participants with realistic, hypothetical shopping situations that mimic real-life decision-making contexts. Participants are asked to respond as they would in actual shopping environments, allowing researchers to assess their spontaneous purchasing tendencies in a controlled setting. The diversity in these choices corresponds to different levels of impulsive consumption.

To validate the effectiveness of the situational simulation method for measuring impulsive consumption, a random sampling approach was adopted. A total of 35 participants were selected to partake in a pilot study, from which 35 valid samples were collected. Following the method described by Rook and Fisher (1995), four simulated shopping scenarios were devised. These included: purchasing a necessary computer or a leisure-oriented gaming console (Scenario 1); buying a suit required for a meeting or a long-desired ring (Scenario 2); purchasing essential milk or buying additional discounted items (Scenario 3); financing a car purchase (Scenario 4). Participants were asked to rate each of the five potential shopping choices in each scenario on a scale from 1 to 5, where higher ratings indicated a higher perceived level of impulsive consumption. For example, in Scenario 2, the option “Not at all tempted by the necklace, only buy the suit” was rated as 1 in impulsive consumption, while “Buy the long-desired necklace and not the suit, wear a shirt or borrow a friend’s suit for the meeting tomorrow” was rated as 5.

To verify that the five shopping options designed for each scenario reflect differences in participants’ levels of impulsive consumption, repeated measures ANOVA was conducted (Table 1). The analysis of variance for the impulsive consumption scores of the five shopping options in each scenario, along with post-hoc comparisons of the scores for the five options within each scenario, revealed significant differences (*p* < 0.05). These results indicate that the five options effectively reflect the differences in impulsive consumption levels among participants in each scenario. After validating the differences in impulsive consumption levels within each scenario, participants’ scores across all four scenarios were aggregated to form a total impulsive consumption score for each individual, which was used as the dependent variable in subsequent analyses.

**Table 1:** One-way repeated measures ANOVA of individual perceptions of impulsive consumption across five shopping choices.

Consumption scenario	SS	*df*	*MS*	*F*	*LSD*
Consumption scenario 1	362.83	4	90.71	937.00^***^	1 < 2 < 3 < 4 < 5
Consumption scenario 2	376.72	4	94.18	724.46^***^	1 < 2 < 3 < 4 < 5
Consumption scenario 3	345.91	4	86.48	713.15^***^	1 < 2 < 3 < 4 < 5
Consumption scenario 4	360.14	4	90.03	937.36^***^	1 < 2 < 3 < 4 < 5

^*^p < 0.05, ^**^p < 0.01, ^***^p < 0.001.

### 2.4 Research procedure

Before the experiment began, participants read the instructions and signed an informed consent form. They were then randomly assigned to either the social resource scarcity priming group or the control group, and received the corresponding questionnaire materials. After reading the questionnaire materials, participants were given unrelated tasks, such as arithmetic problems, to divert their attention from guessing the purpose of the experiment. Participants then answered questions related to the assigned topics. Subsequently, they completed a simulated consumption task and filled out related questionnaires; finally, participants completed the Early Environmental Unpredictability Scale and provided basic personal information, marking the end of the experiment.

### 2.5 Statistical analysis

All statistical analyses were conducted using SPSS 26.0. To assess potential common method bias, Harman’s single-factor test was performed.

Descriptive statistics and Pearson correlation analyses were used to examine the relationships among early environmental unpredictability, perceived resource scarcity, and impulsive consumption. To test the direct effect of early environmental unpredictability on impulsive consumption, a hierarchical regression analysis was conducted. Gender, household registration, education level, and perceived resource scarcity were included as control variables (dummy-coded as needed). Continuous predictors were standardized. Multicollinearity was checked using VIF values, which were all close to 1. A 2 (early environmental unpredictability: high vs. low) × 2 (perceived resource scarcity: scarcity vs. normal) factorial ANOVA was conducted to examine interaction effects. Participants were grouped based on a mean split of unpredictability scores. Simple effect analyses were used to further explore significant interactions. All tests were two-tailed with significance levels set at *p* < 0.05. Effect sizes (η^2^) were reported for ANOVA results.

## 3 Results

### 3.1 Test for common method bias

In this experiment, the collection of data on early environment, perception of environmental resources, and impulsive consumption was conducted through questionnaires within the same simulated scenarios, which could potentially lead to common method bias. To address this issue, Harman’s single-factor test was employed. Analysis revealed that the first factor accounted for 28.77% of the variance, which is below the critical threshold of 40%, suggesting that a serious common method bias did not exist.

### 3.2 Descriptive statistics and correlation analysis of key variables

As shown in Table 2, the descriptive statistics and correlation analysis of the main variables are as follows: impulsive consumption was significantly positively correlated with early environmental unpredictability (*r* = 0.47, *p* < 0.001) and perception of resource scarcity (*r* = 0.23, *p* < 0.001).

**Table 2:** Descriptive statistics and correlation analysis of key variables.

Variable	*M*	*SD*	1	2	3
1 Early environmental unpredictability	36.47	8.05	1		
2 Perception of resource scarcity	17.58	3.73	0.14	1	
3 Impulsive consumption	9.88	3.00	0.47^***^	0.23^***^	1

^*^p < 0.05, ^**^p < 0.01, ^***^p < 0.001.

### 3.3 Direct impact of early environmental unpredictability on impulsive consumption behavior

To explore the relationship between early environment and impulsive consumption, regression analysis was conducted with impulsive consumption as the dependent variable. The first layer of independent variables included demographic variables such as gender, household registration, and education level, as well as the degree of perception of resource scarcity, all of which were dummy-coded prior to inclusion in the model. Early environmental unpredictability and perception of resource scarcity were standardized. There was no severe multicollinearity issue (VIF values were around 1). In Model 1, gender and perception of resource scarcity impacted the level of impulsive consumption. However, in Model 2, after controlling for the aforementioned variables, the impact of early environmental unpredictability on impulsive consumption was significant (unstandardized *B* = 0.40, *t* = 5.35, *p* < 0.001), with other control variables not reaching statistical significance. This is consistent with Hypothesis 1 of the study, with specific results presented in Table 3.

**Table 3:** Regression models of the impact of early environmental unpredictability on impulsive consumption.

Predictor	Impulsive consumption	
	Model 1	Model 2
Gender	−0.18^*^	−0.11
Only-child status	−0.15	−0.06
Household registration type	−0.16	−0.12
Education level	−0.01	0.05
Perception of resource scarcity	0.22^**^	0.18^*^
Early environmental unpredictability		0.40^***^
*F*	5.20^**^	9.87^***^
*_R_2*	0.14	0.28
Δ*R^2^*	0.14	0.14

^*^p < 0.05, ^**^p < 0.01, ^***^p < 0.001.

### 3.4 The impact of early environmental unpredictability on impulsive consumption under conditions of scarcity activation

Experiment 1 was a 2 × 2 factorial design. It referenced the criteria for categorizing high versus low early socioeconomic status (Kong, 2021). Early environmental unpredictability was measured with a mean score (*M* = 36.47, SD = 8.05). Participants scoring above this mean were classified into the high unpredictability group (*n* = 87), while those scoring below were placed in the low unpredictability group (*n* = 74). The independent variables were levels of early environmental unpredictability (high vs. low) and current perceptions of resource scarcity (scarcity vs. normal). The dependent variable was the level of impulsive consumption in a simulated scenario. Descriptive statistics and a factorial ANOVA were conducted, with results shown in Table 4:

**Table 4:** Impulsive consumption levels across four groups.

Early environmental unpredictability	Perception of scarcity		Perception of normal resources	
	*M*	*SD*	*M*	*SD*
High unpredictability group	12.70 (*n* = 41)	3.26	10.02 (*n* = 46)	2.81
Low unpredictability group	9.27 (*n* = 33)	2.67	8.43 (*n* = 41)	1.86

^*^p < 0.05, ^**^p < 0.01, ^***^p < 0.001.

The main effect of early environmental unpredictability was significant, *F*(1, 160) = 35.40, *p* < 0.001, η^2^ = 0.18. This result indicates a significant difference in impulsive consumption levels between subjects with high (*M* = 11.12) and low (*M* = 8.85) early environmental unpredictability. Additionally, the main effect of the manipulation of perceived resource scarcity was significant, *F*(1,160) = 17.40, *p* < 0.001, η^2^ = 0.10. This indicates that subjects perceiving scarcity (*M* = 10.66) demonstrated significantly higher impulsive consumption levels compared to those perceiving abundant resources (*M* = 9.22), showing that impulsive consumption increased following the manipulation of resource scarcity perception.

The interaction between early environmental unpredictability and current perceived resource scarcity was significant, *F*(1,160) = 4.73, *p* < 0.05, η^2^ = 0.03. Simple effects analysis revealed that in the high unpredictability group, subjects perceiving scarcity (*M* = 12.70) exhibited higher impulsive consumption levels than those perceiving normal resources (*M* = 10.02), *F*(1,73) = 18.16, *p* < 0.001, η^2^ = 0.10. Conversely, in the low unpredictability group, there was no significant difference in impulsive consumption levels between subjects perceiving scarcity (*M* = 9.27) and those perceiving normal resources (*M* = 8.43), *F*(1,86) = 2.23, *p* > 0.05, η^2^ = 0.01. The specific interaction effects are illustrated in Table 5.

**Table 5:** ANOVA of early environmental unpredictability and current perceived resource scarcity on impulsive consumption.

Variable	*F*	*p*	*η^2^*
Early environmental unpredictability	35.40	0.00	0.18
Perceived social resource scarcity	17.40	0.00	0.10
Early environmental unpredictability * Perceived social resource scarcity	4.73	0.03	0.03

## 4 Discussion

This study employed the social resource scarcity activation paradigm and explored the relationship between early environmental unpredictability and impulsive consumption through simulated scenarios. The findings suggest that early environmental unpredictability can positively predict impulsive consumption behavior. Specifically, individuals with higher levels of unpredictability in their early environment exhibited greater tendencies toward impulsive consumption, especially when perceiving current resources as scarce.

Firstly, the unpredictability of the early environment can positively predict our impulsive consumption behavior. This is because higher life stress and unpredictability in the early environment make it more likely for individuals to display impulsive behaviors as they grow up. This phenomenon is consistent with previous research findings that early life environmental stress can positively predict impulsive behavior (Griskevicius et al., 2011; Mittal and Griskevicius, 2014). Among demographic variables, gender has been found to predict impulsive consumption, with females showing a higher tendency toward impulsive purchases than males. This might be due to women generally possessing stronger purchasing power in everyday life (Tifferet and Herstein, 2012). However, when the unpredictability of the early environment is included in the model, the impact of gender on impulsive consumption becomes non-significant. This indicates that although gender influences impulsive consumption behavior to some extent, the unpredictability of the early environment plays a more crucial role. This may be because individuals in uncertain environments are more inclined to adopt strategies of immediate gratification to cope with potential resource scarcity or existential threats, a strategy that begins to form in childhood and influences consumption behavior into adulthood. Thus, understanding the impact of the early environment on individual behavior, and how these factors operate across different genders, is crucial for explaining the complexity of impulsive consumption behavior. This not only aids in a deeper theoretical exploration but also provides a basis for developing more effective interventions for consumer behavior. Additionally, while the influence of gender may be diminished under certain conditions, its role in consumer behavior should not be overlooked and warrants further exploration and validation in future research to reveal underlying psychological mechanisms and social factors.

Secondly, the perception of social resource scarcity can predict impulsive consumption behavior. When individuals perceive resources as scarce, they typically score higher on tests of impulsive consumption. This aligns with the sensitization model, which suggests that individuals from varying backgrounds of early environmental unpredictability exhibit significant differences in behavior under conditions of adversity and non-adversity. Specifically, those who grew up in unstable environments may be more inclined to adopt immediate gratification consumption strategies when faced with resource scarcity. This behavior can be seen as an adaptive response, aimed at rapidly utilizing limited resources to ensure short-term survival and satisfaction. The findings from Model 2 indicate that when considering the core variable of early environmental unpredictability, the impact of perceived social resource scarcity on impulsive consumption is significantly reduced. This implies that the unpredictability of the early environment may largely determine an individual’s perception of resource scarcity, and in turn, influence their consumption behavior. This discovery underscores the important role of the early environment in shaping individual psychological and behavioral patterns. Although the perception of social resource scarcity remains a significant factor influencing impulsive consumption, its influence is diminished when early environmental factors are considered. This may be because the unpredictability experienced early in life makes individuals more sensitive to resource scarcity in adulthood, thereby driving them toward more impulsive consumption behaviors. This phenomenon not only provides a new perspective for understanding impulsive consumption behavior but also offers a theoretical basis for developing targeted intervention measures. Future research could further explore the interaction between early environments and the perception of resource scarcity, as well as how improvements in early environments might reduce the occurrence of impulsive consumption behaviors.

Finally, the study revealed that subjects with varying levels of unpredictability in their early environments exhibited different patterns of impulsive consumption when triggered by resource scarcity. Specifically, the higher the unpredictability of the early environment, the more pronounced the tendency toward impulsive consumption in the presence of perceived resource scarcity. This aligns with the sensitization model proposed within life history theory, which suggests that early life conditions predispose individuals to respond differently to adversities encountered later in life. These models indicate that while adults from different childhood environments may behave similarly under benign and non-threatening conditions, their behaviors under adversity can differ significantly. Moreover, research by Griskevicius has also confirmed this, showing that without cues of economic uncertainty, individuals, regardless of their early economic status, do not differ in their impulse to save for the future; differences only arise when cues of resource scarcity are present. Thus, the unpredictability of the early environment not only affects the adaptive mechanisms during an individual’s growth but also profoundly influences their behavioral patterns when facing economic challenges as adults. Triggered by resource scarcity, those who have experienced unstable early environments may be more inclined to adopt strategies maximizing short-term benefits, manifesting as impulsive purchasing behaviors. This behavior is widely discussed in psychology and behavioral economics as a strategy to optimize survival and reproduction opportunities in environments with limited resources. However, in the modern consumer environment, this strategy may no longer be optimal as it can lead to economic decision-making errors, such as excessive debt and insufficient savings. Therefore, understanding the roots and mechanisms of this behavioral pattern is crucial for designing effective economic decision-making education and behavioral intervention strategies. By providing education and social support, helping these individuals develop more robust economic behavior strategies could be key to enhancing societal economic welfare. Specifically, given our findings that individuals with high early environmental unpredictability exhibit stronger impulsive consumption under resource scarcity, targeted interventions could include financial literacy programs tailored to address short-term gratification tendencies, focusing on long-term planning and budgeting skills. Additionally, psychological counseling or support groups could help individuals recognize and mitigate the influence of early environmental stressors on current consumption behaviors, fostering adaptive coping mechanisms during perceived scarcity. Considering the differences in individuals’ early experiences and psychological adaptation mechanisms in policy formulation and personal behavior guidance can more effectively promote healthy economic behavior development and reduce socioeconomic inequality. Such policies might involve community-based initiatives that provide resources and education to at-risk populations with histories of early unpredictability, aiming to break the cycle of impulsive economic decisions driven by past adversities.

Moreover, it is important to note that in this study, early environmental unpredictability was treated as a categorical variable, and a dichotomization (high vs. low) approach was adopted to simulate distinct early-life environmental contexts. This method was chosen to enhance the interpretability of interaction effects and is consistent with the theoretical framework of life history theory, which emphasizes contrasts between environmental conditions. However, we acknowledge that dichotomizing continuous variables may lead to information loss, reduced statistical power, and possibly artificial effects (Altman and Royston, 2006; MacCallum et al., 2002). Therefore, we recognize this as a limitation of the current study and recommend that future research, with larger samples and complete datasets, employ continuous-variable modeling approaches (e.g., GLM or regression analysis) to further test the interaction effects and improve the precision and robustness of the findings.

In conclusion, this study delved into the relationship between early environmental unpredictability and impulsive consumption behavior through the use of scarcity priming paradigms and simulated scenarios. The results show a positive correlation between early environmental unpredictability and impulsive consumption behavior in adulthood, particularly pronounced under conditions of perceived resource scarcity. These findings not only enrich the theory of consumer behavior but also provide a new perspective for understanding actual consumption behavior. Unlike previous studies, this research highlights the impact of the early environment on tendencies toward impulsive consumption and suggests that stability in children’s early environments should be emphasized in family education and social policies to reduce future irrational consumption. Moreover, maintaining a rational consumption attitude in the face of resource scarcity challenges is crucial. Promoting rational consumption concepts and enhancing consumer education during times of resource constraints are vital for constructing a stable and harmonious societal environment. Overall, this study provides new theoretical support for understanding the psychological mechanisms behind impulsive consumption and offers guidance for promoting healthy consumer behavior development.

## 5 Conclusion

This study, employing scarcity priming paradigms and simulated scenarios, has successfully revealed a positive correlation between unpredictability in early environments and impulsive consumption behaviors in adulthood. The results indicate that individuals who experienced unstable early environments are more prone to exhibit impulsive consumption behaviors under conditions of perceived resource scarcity. Specifically, this relationship becomes more pronounced under conditions where resource scarcity is perceived, suggesting that external pressures from resource environments can trigger or exacerbate individuals’ tendencies toward impulsive consumption. Furthermore, compared to individuals from relatively stable early environments, those with higher unpredictability in their early environments demonstrate more frequent and significant impulsive consumption behaviors when faced with resource scarcity. This finding aligns with previous research, which has suggested that instability in early environments may lead to less rationality and more impulsivity in resource management and consumption decisions. These results provide a new perspective on the psychological foundation of individual consumption behaviors and emphasize the critical role of early environments in shaping consumption behavior patterns. Through these findings, we gain a better understanding of how individuals respond to uncertain and unstable environmental influences through impulsive consumption under conditions of perceived resource scarcity. This theoretical contribution expands our understanding of the psychological mechanisms behind impulsive consumption behaviors and provides a foundation for further experimental and theoretical research.

## Data Availability

The raw data supporting the conclusions of this article will be made available by the authors, without undue reservation.
